# ﻿*Nigromargaritatarda* gen. et sp. nov. and distribution of an intron position class within *Pleosporales*

**DOI:** 10.3897/imafungus.16.145425

**Published:** 2025-02-28

**Authors:** Meng-Yuan Li, Xiang Sun, Yu-Qing Liu, Sheng-Hui Qin, Min Li, Xue-Li He

**Affiliations:** 1 School of Life Sciences, Hebei University, Baoding 071002, China Hebei University Baoding China; 2 Engineering Research Center of Ecological Safety and Conservation in Beijing-Tianjin-Hebei (Xiong’an New Area) of MOE, Baoding 071002, China Engineering Research Center of Ecological Safety and Conservation in Beijing-Tianjin-Hebei (Xiong’an New Area) of MOE Baoding China; 3 College of Biology and Food, Shangqiu Normal University, Shangqiu 476000, China Shangqiu Normal University Shangqiu China

**Keywords:** Dark septate endophyte, intron, molecular phylogeny, position class, taxonomy, *Trematosphaeriaceae*

## Abstract

*Pleosporales*, the largest order in *Dothideomycetes*, has a broad host range and inhabits host plants as epiphytes, endophytes, parasites and saprophytes. *Trematosphaeriaceae* is a monophyletic family in *Pleosporales*, composed of species of deviated ecological background and morphological traits. In this study, we described a new fungal taxon under *Trematosphaeriaceae*, based on root endophytic fungi recovered from the desert plant *Gymnocarposprzewalskii* in Gansu Province, China. The taxon is characterised by simple, aseptate conidia and pycnidia in unusually small sizes. Multilocus phylogenetic analysis, based on ITS, LSU, SSU and TEF sequences and a morphology study indicated that the taxon represented a new genus within the *Trematosphaeriaceae* and was named *Nigromargaritatarda*. Intriguingly, an intron of 355 bp in length located at site 453 on the ribosomal SSU gene was detected in one strain of *N.tarda*. Sequence analysis and phylogenetic analysis indicated that the intron belongs to an intron position class (Pcl) restricted to *Pleosporales*. Phylogeny affiliated distribution of this Pcl was confined at the genus or lower level, suggesting a horizontal transmission pattern of this Pcl. This study established a new genus in *Trematosphaeriaceae* and depicted the spread features of a less-documented Pcl amongst *Pleosporales* families with high resolution, which promotes our understanding of the origin and transmission mechanism of such mobile genetic elements.

## ﻿Introduction

*Pleosporales* is the largest order within the class *Dothideomycetes* (Zhang et al. 2012). It is characterised by perithecioid ascomata, usually with a papillate apex; ostioles with or without periphyses; the presence of cellular pseudoparaphyses; bitunicate and fissitunicate asci; and variously shaped, aseptate or septate ascospores, with asexual forms of coelomycetes and hyphomycetes (Zhang et al. 2012; Hyde et al. 2013; Sun et al. 2022; Yu et al. 2022). Members of *Pleosporales* are globally distributed and are commonly found in diverse terrestrial, marine and freshwater habitats (Jones et al. 2020; Li et al. 2021b; Hu et al. 2023). They are widely represented as epiphytic, endophytic, saprophytic and parasitic fungi on host plants (Mapook et al. 2016; Matsumura et al. 2018; Ferdinandez et al. 2021). *Pleosporales* is one of the most representative groups of plant-associated fungi in arid and semi-arid regions (Knapp et al. 2015). A previous study indicated that a significant proportion of the endophytic fungi isolated from desert plants, especially in roots, belong to *Pleosporales* (Zuo et al. 2022).

*Trematosphaeriaceae* is a family within *Pleosporales* that currently includes seven genera. According to the online database IndexFungorum and MycoBank, six of these genera are recognised: *Emarellia*, *Falciformispora*, *Fuscosphaeria* (monotypic genus), *Halomassarina* (monotypic genus), *Meanderella* (monotypic genus) and *Trematosphaeria* (MycoBank , Index Fungorum 2024). *Hadrospora*, initially proposed within the family *Phaeosphaeriaceae* (Boise 1989), has been a subject of debate regarding its placement within the family, based on its morphological characteristics (Zhang et al. 2012) and was eventually tentatively placed in *Trematosphaeriaceae* (Phookamsak et al. 2014; Pem et al. 2019). The family *Trematosphaeriaceae* was originally introduced to accommodate three plant-associated, ascomata produced genera: *Falciformispora*, *Halomassarina* and *Trematosphaeria* (Kohlmeyer and Volkmann-Kohlmeyer 1987; Hyde 1992; Suetrong et al. 2009; Suetrong et al. 2011). Nevertheless, the subsequent additions to the family did not strictly adhere to the morphology criteria and exhibited distinct ecological distribution. The inclusion of *Hadrospora* was primarily driven by morphological consideration in the absence of sequence data (Zhang et al. 2012; Phookamsak et al. 2014). The three additional genera, namely, *Emarellia*, *Meanderella* and *Fuscosphaeria* are sterile fungi and were placed in *Trematosphaeria*, based on molecular phylogenetic evidence (Borman et al. 2016; Pintye and Knapp 2021; Ahmed et al. 2022). *Emarellia* and *Meanderella* are human dermatophytes, *Fuscosphaeria* is a root-colonising fungus, while *Hadrospora* has been recovered from wood of various plants in both terrestrial and freshwater habitats. Thus, *Trematosphaeriaceae* represents an ecologically deviated clade composed of teleomorphic and sterile fungi within *Pleosporales*.

Introns are non-expressed nucleotide sequences within a gene. They are widely present in eukaryotic genomes and are removed during transcription (Gilbert 1978). In fungi, introns are found in both fungal and mitochondrial genomes, where they evolve and play significant roles in gene regulation and alternative splicing processes. However, research on introns within fungal genomes remains limited and many aspects of their origin and evolution are still poorly understood. Several hypotheses have been proposed regarding the origin of introns in fungal genomes. For instance, Stajich et al. (2007) proposed the existence of “intron-rich ancestors” in fungi, suggesting that fungal genomes were historically abundant in introns, with extensive intron loss occurring across various fungal clades throughout their evolutionary history. Megarioti and Kouvelis (2020) argued that introns, particularly in mitochondrial genomes, may have originally been selfish genetic elements that proliferate across genomes via mechanisms such as horizontal gene transfer (HGT), with introns frequently carrying homing endonucleases that promote their own propagation. Another hypothesis posits that introns may have originated from endosymbiotic bacterial ancestors and then diversified within fungal lineages (Wu et al. 2017). Novel mechanisms, such as the multiplication of introner-like elements, have also been identified as significant contributors to intron gains in fungi (Burgt et al. 2012). The dynamic nature of intron loss and gain has been highlighted as a key aspect of fungal genome evolution, with some introns being lost over time, while others are gained through various mechanisms, including horizontal transfer from other species (Lim et al. 2021).

In fungi, introns have been identified in the 18S ribosomal RNA genes. Cubero et al. (2000) were the first to report the presence of multiple spliceosomal introns within ribosomal genes. These introns are typically located in highly-conserved regions of the 18S gene and exhibit variation in size, sequence and position across different fungal species. Interestingly, they are often conserved across closely-related species, suggesting an ancient origin and potential functional importance. Bhattacharya et al. (2000) suggested that introns are of relatively recent origin and are flanked by non-random sequences (e.g. AG-intron-G, as described in their research). Nevertheless, the presence of introns can vary between closely-affiliated species; for example, in *Scytalidiumdimidiatum* and *S.hyalinum*, differences were shown in intron presence (Machouart et al. 2004), which may contribute to species differentiation. Investigating intron distribution across the phylogenetic lineage of fungal species facilitates our comprehension of the patterns and factors influencing fungal genome evolution and intron coevolution. Nonetheless, research on introns has historically been limited, particularly in terms of their phylogenetic associations.

In our study of endophytic fungi in plants from the arid desert regions of northwest China, two fungal isolates were consecutively recovered from the healthy roots of *Gymnocarposprzewalskii* in Minqin County, Gansu Province. Morphological and molecular evidence confirmed that the isolates represent a new genus within the family *Trematosphaeriaceae*, *Pleosporales*. In addition, we observed that one isolate carried an intron in the 18S gene, while the other did not. In this study, we describe and establish a new genus within the *Trematosphaeriaceae* family and propose a distribution pattern for the intron position class (Pcl) to which it belongs within the order *Pleosporales*. Our study will provide new insights and evidence for understanding the mechanisms of intron transmission amongst fungal species.

## ﻿Materials and methods

### ﻿Strains and morphological study

In a previous study, strains H331 and H263 were isolated as dark septate endophytes (DSEs) from the healthy roots of *Gymnocarposprzewalskii* in the Minqin National Reserve (38°59'48"N, 103°2'33"E), Gansu Province, China, in September 2019 and July 2020, respectively (Li et al. 2021a). The fungal isolates were stored at - 80 °C, then transferred on to PDA, OA and MEA media and incubated in the dark at 27 °C for at least 28 days prior to morphological observations of colonies and microscopic features. The colours, texture and pigmentation of the colonies were recorded and their diameters were measured on the 7^th^ and 28^th^ days. Fresh mycelia were mounted in sterile double-distilled water and observed under a microscope (Nexcope NE900, Zhejiang, China). When necessary, frozen sections of microscopic structures were cut with a cryostat (CM1860, Leica, Germany). The sizes of the conidiogenous cells and conidia were measured at least 20 times. Specimens of the oven-dried cultures were preserved at the Herbarium of Mycology, Chinese Academy of Sciences (HMAS). Living cultures of all strains were deposited in the China General Microbiological Culture Collection Center (CGMCC), Beijing, China.

### ﻿DNA extraction, PCR amplification and sequencing

DNA was extracted using an alkaline thermolysis method. A small amount of fresh mycelium, visible to the naked eye, was picked from the margins of each colony and placed in a well of a 96-well PCR plate. To each well, 50 µl of sodium hydroxide (NaOH) solution (10 µmol/l) was added and the plate was then placed in a thermal cycler and incubated at 98 °C for 20 min. Sequence data for the ITS, LSU, SSU and TEF regions were generated in this study. The ITS region was amplified using the primer pair ITS4 and ITS5 (White et al. 1990), the LSU gene was amplified using LR0R and LR5 (Vilgalys and Hester 1990), the SSU region was amplified using NS1 and NS4 (White et al. 1990) and the TEF region was amplified using EF1-983F and EF1-2218R (Yang et al. 2019). All PCR reactions were performed in an EASTWIN™ Thermal Cycler (ETC811, Suzhou, China) with a 25 µl reaction system containing 12.5 µl of 2× buffer, 3 µl of template DNA, 1 µl of each primer and 7.5 µl of sterilised double-distilled H2O. The thermal cycling conditions for each locus are provided in Table 1. The PCR products were examined by electrophoresis at 120 V for 30 min in a 1.2% (w/v) agarose gel in 1× TAE buffer, then visualized under ultraviolet light after staining with ethidium bromide. The PCR products were sent to General Biology Company (Anhui, China) for Sanger sequencing. The sequences were reviewed and manually modified with Chromas v.1.0.1.1 to remove low-quality base calls from both ends. The modified sequences were subsequently deposited in GenBank.

**Table 1.** PCR amplification procedure for each gene fragment.

Gene fragment	Ampliﬁcation conditions
SSU and LSU	Predenaturation at 94 °C for 5 min; denaturation at 94 °C for 1 min, annealing at 55 °C for 1 min, extension at 72 °C for 1 min, 35 cycles; Extension at 72 °C for 10 min.
TEF	Predenaturation at 94 °C for 5 min; denaturation at 94 °C for 50 s, annealing at 51 °C for 1 min, extension at 72 °C for 1 min, 35 cycles; Extension at 72 °C for 8 min.
SSU cDNA	Predenaturation at 95 °C for 3 min; denaturation at 95 °C for 30 s, annealing at 55 °C for 30 s, extension at 72 °C for 45 s, 35 cycles; Extension at 72 °C for 5 min.

### ﻿﻿Molecular phylogenetic analysis of *Nigromargaritatarda*

Preliminary alignments and BLAST suggested that both H331 and H263 were highly similar in sequence and might represent a novel clade within *Pleosporales*. Representative sequences from *Pleosporales* members, particularly those from type materials, were selected for subsequent phylogenetic analysis to determine the taxonomic placement of H331 and H263 placement (Table 2, Please refer to Suppl. material 1 for detailed information and alignment matrices for all datasets). Multiple sequence alignment was performed using MAFFT v. 7.3.13. The alignments were reviewed in BioEdit v.7.0.9 to trim both ends and manually correct any obvious misalignments (Suppl. material 2: Align_combine_60). In this study, a 355 bp intron was identified in the SSU of strain H263 and was manually excised. The combined LSU, SSU, ITS and TEF sequence datasets were then analysed using Maximum Parsimony (MP), Maximum Likelihood (ML) and Bayesian Inference (BI) methods using PAUP v. 4.0, RAxML v. 8.2.12 and MrBayes v. 3.2.6, respectively (Suppl. material 2: comb60_PAUP.nxs, comb60_MrBayes.nxs). *Dothideasambuci* (DAOM 231303) and *Phaeoscleradematioides* (CBS 157.81) in *Dothideales* were selected as outgroups, based on Ahmed et al. (2014).

The optimal models for phylogenetic analysis were predicted using MrModelTest v. 2.4, based on the Akaike Information Criterion (AIC). MP and BI analyses were performed using the GTR+I+G model and ML was calculated using the GTRGAMMA model. Both MP and ML were conducted with 1,000 bootstraps. In the BI analysis, eight Markov chains were run for 1,000,000 generations, with trees sampled every 100 generations. The first 25% of the trees were discarded as burn-in and the remaining trees were used to compute the Bayesian Posterior Probabilities (BPPs). The phylogenetic trees were visualised and edited with FigTree, TreeView and Adobe Illustrator.

### ﻿RNA extraction and cDNA amplification

Reverse transcription PCR (rtPCR) was conducted to acquire the cDNA of the 18s partial gene in this study. RNA was extracted from fresh mycelia using the RNAprep Pure Cell/Bacteria Kit (cat. DP430, TIANGEN BIOTECH, Beijing, China) following the manufacturer’s instructions. The template RNA-primer mixture was prepared by mixing 1 μg of total RNA, 1.0 μl of random hexamer primer and adjusting the final volume to 13.5 μl with RNase free ddH2O on ice. The mixture was incubated at 70 °C for 2 min and then placed immediately on ice. RNA was reversely transcribed using the PrimeScriptTM 1^st^ strand cDNA Synthesis Kit (Takara Bio, Japan). The following components were added to the 13.5 μl of the RNA-primer mixture on ice: 4.0 μl of 5×Reaction Buffer, 0.5 μl of Recombinant RNase Inhibitor, 1.0 µl of MMLV Reverse Transcriptase and 1.0 µl of dNTP Mix (10 mM each). The mixture was gently shaken, incubated at 42 °C for 60 min, then heated at 94 °C for 5 min to stop the reaction and subsequently placed on ice for further processing.

The cDNA templates were amplified using specific primer pairs designed for this study (F1, 5’-CGATACGGGGAGGTAGTGAC-3’; R1, 5’-TTCTCCAGGAAAGAAGGCCC-3’). The amplifications were performed in an ABI thermal cycler (ABI-2720, Applied Biosystems, USA) with a 25 µl reaction system containing 2.0 μl of cDNA template, 1.25 μl of forward and reverse primer (10 μM each), 12.5 μl of Q5 High-Fidelity 2× Master Mix and 8.0 μl of nuclease-free water. The PCR products were examined as described above. The PCR products were sent for sequencing using the Sanger method at Personalbio Technology Company (Shanghai, China). The sequences were reviewed and modified as described above and deposited in GenBank.

### ﻿Dataset preparation for intron analysis

The 355 bp intron present in the 18S gene strain H263 was subjected to BLAST in GenBank, using the “Core nucleotide database (core_nt)” within the “Standard databases (nr etc.)” for all organisms, with uncultured/environmental sample sequences excluded and optimised for highly-similar sequences using the megablast algorithm. The searching identified 464 references. The 18S sequences of H263 and 464 references were aligned, manually modified and the 18S gene regions were excised using MAFFT and BioEdit, producing an alignment dataset consisting only of introns from 465 18S genes (Suppl. material 2: Align_onlyintron_465, Info_onlyintron_465). In addition, 287 references with alignment scores higher than 200 were selected for subsequent intron phylogenetic analysis. The 18S sequences from H263 and 253 references were aligned, manually modified and trimmed using MAFFT and BioEdit, as described above, for phylogenetic analysis. Identical sequences were removed and the modified alignments of 254 sequences (Suppl. material 2: Align_all_254) were divided into two datasets: one containing only intron sequences from the 18S gene of H263 and 253 references (Suppl. material 2: Align_onlyintron_254) and another containing the 18S exon sequences with introns removed (Suppl. material 2: Align_nointron_254).

The variation rates of nucleotide bases in the 18S genes carrying introns were calculated using the dataset nointron_254. Sequence lacking either the left or right flank of the intron were excluded, resulting in an alignment dataset with 229 sequences (Suppl. material 2: Align_nointron_229, Info_nointron_229).

The ratios of intron carriers to non-carriers within specific species were calculated, based on available data deposited in GenBank. References without species information (denoted as “sp.”) were excluded from the 254 references, which encompassed sequences from 126 fungal species. For each species, all its 18S sequences were retrieved from GenBank using the following search strategy: “SPECIES NAME” [Organism] AND (18s [All Fields] OR “small subunit ribosomal” [All Fields]) NOT 5.8s [All Fields]. The carrier:non-carrier ratios were calculated for species with more than two 18S gene records (Suppl. material 2: Info_species_ratio).

To investigate the spread of introns within *Pleosporales*, we compiled a dataset for ancestral character state reconstructions (ASRs). This dataset included introns from 125 fungal species (with *Colletotrichumgloeosporioides* excluded) within *Pleosporales* (Suppl. material 2: Align_ASR_onlyintron_125, Info_ASR_onlyintron_125).

### ﻿The co-phylogeny analysis and ancestral character reconstructions for introns

PCoA ordination was performed, based on the dissimilarity matrix of the alignment dataset of onlyintron_465 with the cmdscale function and plotted in R (Suppl. material 2: R_codes_used_in_present_manuscript.r). To assess the evolutionary trajectory of introns within *Pleosporales*, the datasets onlyintron_254, nointron_254, and ASR_onlyintron_125 were subjected to ML analysis as described above for co-phylogeny analysis and ancestral character reconstruction. The best ML trees generated from the onlyintron_254 and nointron_254 datasets were used to perform PACo and parafit analyses to test the co-evolution between introns and the 18S gene that carries them (Suppl. material 2: RAxML_bipartitions.254_onlyintron_ML, RAxML_bipartitions.254_nointron_ML, Info_Co-phylogeny-254). The Procrustes Application to Co-phylogenetic analysis (PACo) was carried out using the PACo function from the R package “paco” (Balbuena et al. 2020) and the parafit analysis was performed using the parafit function from the R package “ape” (Paradis and Schliep 2019). The 125 species carrying introns within *Pleosporales* were coded according to their family placements (Suppl. material 2: Align_ASR_onlyintron_125, Info_ASR_onlyintron_125). Ancestral character estimation was performed using the ace function from the R package “ape”. The phylogenetic trees were visualised using tools available in the R packages “ggtree” and “phytools” (Yu et al. 2017; Revell 2024).

## ﻿Results

### ﻿Phylogenetic analysis

**Figure 1.**
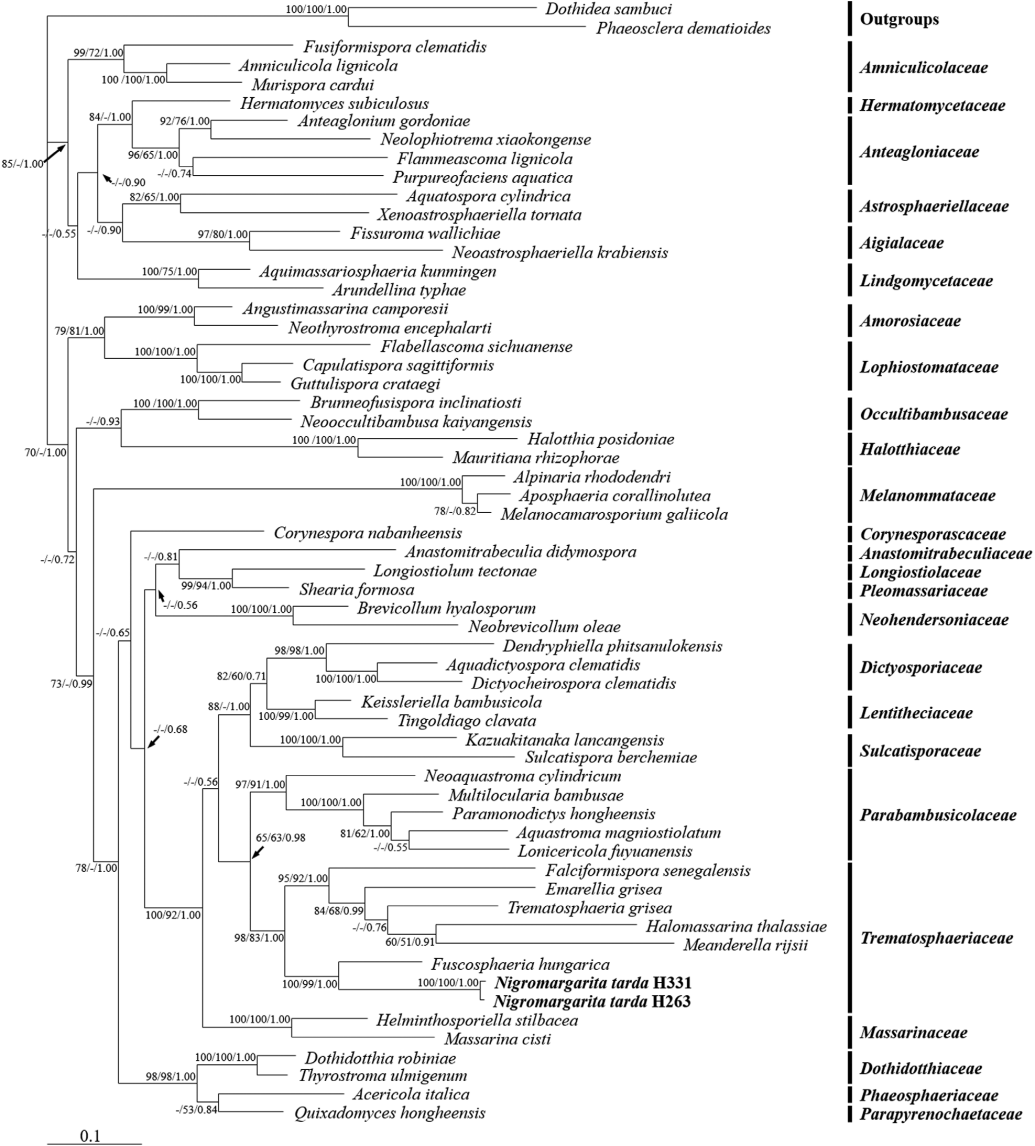
Phylogram of the combined genes of SSU, ITS, LSU and TEF, based on Bayesian analysis. The topology of the Bayesian tree was similar to those generated in the ML and MP analyses (Suppl. material 2: RAxML_bipartitions.comb60_ML, comb60_MPtree.nex, comb60_MrBayes.nxs.con). The numbers at each node represent the percentages of bootstrap values in ML (left) and MP (middle) calculated from 1,000 replications and the BPP (right). - indicates lack of support or support less than 50% (or 0.5 for BPP) for a particular clade. Scale bar indicates 0.1 expected changes per site.

In our preliminary study, BLAST results indicated that strains H331 and H263 likely belong to *Pleosporales*. To determine their precise taxonomic placement, 54 species of 25 families in *Pleosporales* were included in the molecular phylogenetic analysis as reference sequences, with *Dothideasambuci* and *Phaeoscleradematioides* used as outgroups (Table 2). The nucleotide sequences of ITS, LSU, SSU and TEF from various species were retrieved from GenBank, along with those generated in our study. The aligned and trimmed datasets for the four loci, comprising sequences from 58 species across 25 families in *Pleosporales* and two outgroups, were concatenated into a combined dataset of 3,499 bp. This dataset included 947 bp for SSU, 869 bp for ITS, 779 bp for LSU and 904 bp for TEF. Bayesian analysis produced a phylogeny tree with a topology similar to those derived from ML and MP analysis. The Bayesian consensus tree indicated that members of *Trematosphaeriaceae* formed a monophyletic clade with two main branches. Strains H331 and H263 formed a highly-supported clade (ML: 100%; MP: 100%; BI: 1.00; Fig. 1) within the *Trematosphaeriaceae* family (ML: 98%; MP: 83%; BI: 1.00), distinct from its sister clade, *Fuscosphaeria*, which exhibited long branches (ML: 100%; MP: 99%; BI: 1.00).

**Table 2.** The fungal species and GenBank accession numbers of their sequences used in phylogenetic analysis.

Species	Culture/voucher	ITS	LSU	SSU	TEF	Reference
*Nigromargaritatarda*	**H331 T**	PP564858	PP555603	PP564856	PP768963	this study
*Nigromargaritatarda*	**H263**	PP564859	PP555604	PP564857	PP768964	this study
*Fissuromawallichiae*	MFLUCC 15-0315B **T**	NR_174808	NG_079533	NG_077422	MN953046	Konta et al. (2020)
*Neoastrosphaeriellakrabiensis*	MFLUCC 11-0025 **T**	NR_120004	NG_042599	NG_061118	-	Liu et al. (2011)
*Fusiformisporaclematidis*	MFLUCC 17-2077	MT310589	MT214542	MT226661	MT394725	Phukhamsakda et al. (2020)
*Murisporacardui*	MFLUCC:13-0761	KT736082	KT709176	KT709183	KT709190	Wanasinghe et al. (2015)
*Amniculicolalignicola*	CBS:123094	MH863274	MH874798	-	-	Vu et al. (2019)
*Angustimassarinacamporesii*	MFLU 18-0057 **T**	NR_168223	MN244167	MN244173	-	Hyde et al. (2020)
*Neothyrostromaencephalarti*	CPC 35999	MN562105	MN567613	-	MN556831	Crous et al. (2019)
*Anastomitrabeculiadidymospora*	MFLU 20-0694 **T**	NR_172008	MW412978	MW412977	MW411338	Bhunjun et al. (2021)
*Anteagloniumgordoniae*	MFLUCC 17-2431 **T**	NR_163338	NG_066312	NG_065778	MK360042	Jayasiri et al. (2019)
*Flammeascomalignicola*	MFLUCC 10-0128b	KT324582	KT324583	KT324584	KT324585	Ariyawansa et al. (2015)
*Neolophiotremaxiaokongense*	KUMCC 20-0173 **T**	NR_182353	MT957892	MT957899	MT968871	Ren et al. (2021)
*Purpureofaciensaquatica*	MFLUCC 18-1241 **T**	NR_171968	MN913717	-	MT954372	Dong et al. (2020)
*Aquatosporacylindrical*	MFLUCC 18-1287	MT627673	MN913715	MT864327	MT954375	Dong et al. (2020)
*Xenoastrosphaeriellatornata*	MFLUCC 11-0196	-	KT955467	KT955447	KT955429	Phookamsak et al. (2015)
*Corynesporanabanheensis*	HJAUP C2048	OQ060577	OQ060580	OQ060576	OQ067526	Liu et al. (2023)
*Dothidotthiarobiniae*	MFLUCC 16-1175	MK751727	MK751817	MK751762	MK908017	Senwanna et al. (2019)
*Thyrostromaulmigenum*	MFLUCC 16-1166	MK751756	MK751846	MK751791	MK908046	Senwanna et al. (2019)
*Halotthiaposidoniae*	BBH 22481	-	GU479786	GU479752	-	Suetrong et al. (2009)
*Mauritianarhizophorae*	BCC28867	-	GU371825	GU371833	GU371818	Schoch et al. (2009)
*Hermatomycessubiculosus*	MFLUCC 15-0843 **T**	NR_154091	NG_059689	NG_063612	KX259527	Hyde et al. (2016)
*Aquimassariosphaeriakunmingensis*	KUMCC 18-1019	-	MT627661	MT864312	MT954409	Dong et al. (2020)
*Arundellinatyphae*	MFLUCC:16-0310	KX274246	KX274252	KX274257	-	Hyde et al. (2016)
*Longiostiolumtectonae*	MFLUCC 12-0562 **T**	NR_148100	-	NG_061231	-	Doilom et al. (2017)
*Sheariaformosa*	HKAS_107034	OR053810	OQ703056	OQ703050	OQ708655	Madagammana et al. (2023)
*Capulatisporasagittiformis*	HHUF 29754 **T**	NR_119393	NG_042319	NG_060997	-	Tanaka and Hosoya (2008), Hirayama and Tanaka (2011)
*Flabellascomasichuanense*	CGMCC 3.20936 **T**	NR_182595	NG_088357	NG_087913	ON381179	Yu et al. (2022)
*Guttulisporacrataegi*	MFLUCC 13-0442	KP899134	KP888639	KP899125	KR075161	Thambugala et al. (2015)
*Aquadictyosporaclematidis*	MFLUCC 17-2080	MT310592	MT214545	MT226664	MT394727	Phukhamsakda et al. (2020)
*Dendryphiellaphitsanulokensis*	MFLUCC 17-2513	MG754400	MG754401	MG754402	-	Hyde et al. (2018)
*Dictyocheirosporaclematidis*	MFLUCC 17-2089	MT310593	MT214546	MT226665	MT394728	Phukhamsakda et al. (2020)
*Alpinariarhododendri*	MFLU 20-0278	MT229210	MT229208	MT229209	MT254066	Thiyagaraja et al. (2020)
*Melanocamarosporiumgaliicola*	MFLUCC 13-0545	-	OR206417	OR206407	-	Wijayawardene et al. (2016)
*Brevicollumhyalosporum*	MAFF 243400	LC271242	LC271239	LC271236	LC271245	Tanaka et al. (2017)
*Neobrevicollumoleae*	UESTCC:23.0068	OR253106	OR253258	OR253184	-	Li et al. (2023)
*Brunneofusisporainclinatiostiola*	GZCC:21-0185	MZ964867	MZ964876	MZ964885	OK061070	Yu et al. (2021)
*Neooccultibambusakaiyangensis*	GZCC:21-0184	MZ964869	MZ964878	MZ964887	OK061072	Yu et al. (2021)
*Quixadomyceshongheensis*	KUMCC 20-0215	MW264215	MW264194	MW264224	MW256816	Wanasinghe et al. (2021)
*Acericolaitalic*	MFLUCC 13-0609 **T**	NR_156344	NG_057143	NG_063642	-	Phookamsak et al. (2017)
*Mauritianarhizophorae*	BCC28867	-	GU371825	GU371833	GU371818	Schoch et al. (2009)
*Hermatomycessubiculosus*	MFLUCC 15-0843 **T**	NR_154091	NG_059689	NG_063612	KX259527	Hyde et al. (2016)
*Aquimassariosphaeriakunmingensis*	KUMCC 18-1019	-	MT627661	MT864312	MT954409	Dong et al. (2020)
*Arundellinatyphae*	MFLUCC:16-0310	KX274246	KX274252	KX274257	-	Hyde et al. (2016)
*Longiostiolumtectonae*	MFLUCC 12-0562 **T**	NR_148100	-	NG_061231	-	Doilom et al. (2017)
*Sheariaformosa*	HKAS_107034	OR053810	OQ703056	OQ703050	OQ708655	Madagammana et al. (2023)
*Capulatisporasagittiformis*	HHUF 29754 **T**	NR_119393	NG_042319	NG_060997	-	Tanaka and Hosoya (2008), Hirayama and Tanaka (2011)
*Flabellascomasichuanense*	CGMCC 3.20936 **T**	NR_182595	NG_088357	NG_087913	ON381179	Yu et al. (2022)
*Guttulisporacrataegi*	MFLUCC 13-0442	KP899134	KP888639	KP899125	KR075161	Thambugala et al. (2015)
*Aquadictyosporaclematidis*	MFLUCC 17-2080	MT310592	MT214545	MT226664	MT394727	Phukhamsakda et al. (2020)
*Dendryphiellaphitsanulokensis*	MFLUCC 17-2513	MG754400	MG754401	MG754402	-	Hyde et al. (2018)
*Dictyocheirosporaclematidis*	MFLUCC 17-2089	MT310593	MT214546	MT226665	MT394728	Phukhamsakda et al. (2020)
*Alpinariarhododendri*	MFLU 20-0278	MT229210	MT229208	MT229209	MT254066	Thiyagaraja et al. (2020)
*Melanocamarosporiumgaliicola*	MFLUCC 13-0545	-	OR206417	OR206407	-	Wijayawardene et al. (2016)
*Brevicollumhyalosporum*	MAFF 243400	LC271242	LC271239	LC271236	LC271245	Tanaka et al. (2017)
*Neobrevicollumoleae*	UESTCC:23.0068	OR253106	OR253258	OR253184	-	Li et al. (2023)
*Brunneofusisporainclinatiostiola*	GZCC:21-0185	MZ964867	MZ964876	MZ964885	OK061070	Yu et al. (2021)
*Neooccultibambusakaiyangensis*	GZCC:21-0184	MZ964869	MZ964878	MZ964887	OK061072	Yu et al. (2021)
*Quixadomyceshongheensis*	KUMCC 20-0215	MW264215	MW264194	MW264224	MW256816	Wanasinghe et al. (2021)
*Acericolaitalic*	MFLUCC 13-0609 **T**	NR_156344	NG_057143	NG_063642	-	Phookamsak et al. (2017)
*Keissleriellabambusicola*	KUMCC 18-0122	MK995881	MK995880	NG_067715	MN213156	Jiang et al. (2019)
*Tingoldiagoclavata*	MFLUCC 19-0496 **T**	NR_174047	NG_078686	NG_078748	-	Xu et al. (2020)
*Massarinacisti*	CBS 266.62	LC014568	AB807539	AB797249	AB808514	Tanaka et al. (2015)
*Helminthosporiellastilbacea*	MFLUCC 15-0813 **T**	NR_176743	NG_081484	NG_081401	MT928151	Konta et al. (2021)
*Kazuakitanakalancangensis*	HKAS 122922 **T**	NR_177183	NG_081546	NG_081435	ON009263	Wanasinghe et al. (2021)
*Sulcatisporaberchemiae*	KUMCC 21-0823	ON009126	ON009110	ON009094	ON009269	Wanasinghe et al. (2021)
*Lonicericolafuyuanensis*	MFLU 19-2850 **T**	NR_172419	MN917865	MN917867	MN938324	Yasanthika et al. (2020)
*Paramonodictyshongheensis*	KUMCC 21-0343	ON350762	ON329822	ON329821	OL505582	Seldeslachts et al. (2021), Yang et al. (2022)
*Aquastromamagniostiolatum*	HHUF 30122 **T**	NR_153583	NG_056936	NG_061000	-	Tanaka et al. (2015)
*Multiloculariabambusae*	MFLUCC 11-0180 **T**	NR_148099	NG_059654	NG_061229	KU705656	Phukhamsakda et al. (2018)
*Neoaquastromacylindricum*	MFLUCC 19-0489 **T**	NR_176128	MN473054	MN473048	MN481600	Samarakoon et al. (2019)
*Falciformisporasenegalensis*	CBS 196.79 **T**	KF015673	NG_057981	NG_062928	KF015687	Ahmed et al. (2014)
*Meanderellarijsii*	CBS 143917 **T**	NR_175775	NG_081354	NG_081373	-	Ahmed et al. (2022)
*Trematosphaeriagrisea*	CBS 332.50 **T**	NR_132039	NG_057979	NG_062930	KF015698	Ahmed et al. (2014)
*Halomassarinathalassiae*	JK 5262D	-	GU301816	-	GU349011	Schoch et al. (2009)
*Fuscosphaeriahungarica*	DSE883	MW209054	MW209059	-	MW238843	Pintye and Knapp (2021)
*Emarelliagrisea*	NCPF7066 **T**	LT160922	LT160923	-	LT160934	Borman et al. (2016)
*Aposphaeriacorallinolutea*	MFLU:16-2412	MT177916	MT177943	MT177971	MT454004	Li et al. (2020)
*Dothideasambuci*	DAOM 231303 **T**	NR_111220	NG_027611	NG_012432	-	Lutzoni et al. (2004), Schoch et al. (2014)
*Phaeoscleradematioides*	CBS 157.81 **T**	NR_145341	GU301858	GU296184	GU349047	Schoch et al. (2009)

Note: Type materials are labelled with “T”. “-” indicates that the sequence is unavailable.

### ﻿Taxonomy

#### ﻿ Nigromargarita

Taxon classificationAnimalia PleosporalesTrematosphaeriaceae

M.Y. Li & X. Sun gen. nov.

E1DDB2BE-8EDB-53B8-A906-4998F73C9F76

854411

Fig. 2

##### Type species.

*Nigromargaritatarda*.

##### Etymology.

The taxon initially forms small and pigmented colonies in culture resembling a black pearl.

##### Description.

**Teleomorph**: undetermined. **Anamorph**: Conidiomata pycnidial, separated in aerial hyphae, globose, brown to dark-brown, unilocular, thin-walled. Pycnidial walls multi-layered of ***textura angularis*** cells. Conidiogenous cells formed from the inner walls of the pycnidia, determinate, cylindrical to doliiform, monoblastic, hyaline. Conidia obovoid to clavate, hyaline, no septate, smooth-walled.

#### ﻿ Nigromargarita tarda

Taxon classificationAnimalia PleosporalesTrematosphaeriaceae

M.Y. Li & X. Sun sp. nov.

98B15DE7-58D1-58F0-955B-D70CEB218359

854411

Fig. 2

##### Etymology.

Epithet derives from the slow growth rate of colonies on media.

##### Description.

Endophytic on healthy root of *G.przewalskii*. **Teleomorph**: undetermined. **Anamorph**: Hypha-branched, hyaline to brown, septate, thick-walled, verrucous. Conidiomata pycnidial, superficial, separated in aerial hyphae, globose, brown to dark-brown, unilocular, thin-walled, diameter 31–39 μm, not exceeding 50 μm. Pycnidial walls multi-layered of ***textura angularis*** cells. Conidiophore simple. Conidiogenous cells formed from the inner walls of the pycnidia, phialidic, determinate, cylindrical, straight or curved, monoblastic, hyaline, smooth, thin-walled. Conidia obovoid to clavate, hyaline, smooth, non-septate, 2.1–3.6 × 1.7–2.4 μm (x– = 2.93 × 1.95 μm, n = 20).

##### Habitat.

Healthy roots of *Gymnocarposprzewalskii*.

##### Distribution.

Minqin County, Wuwei City, Gansu Province, China.

##### Culture characteristics.

**Figure 2.**
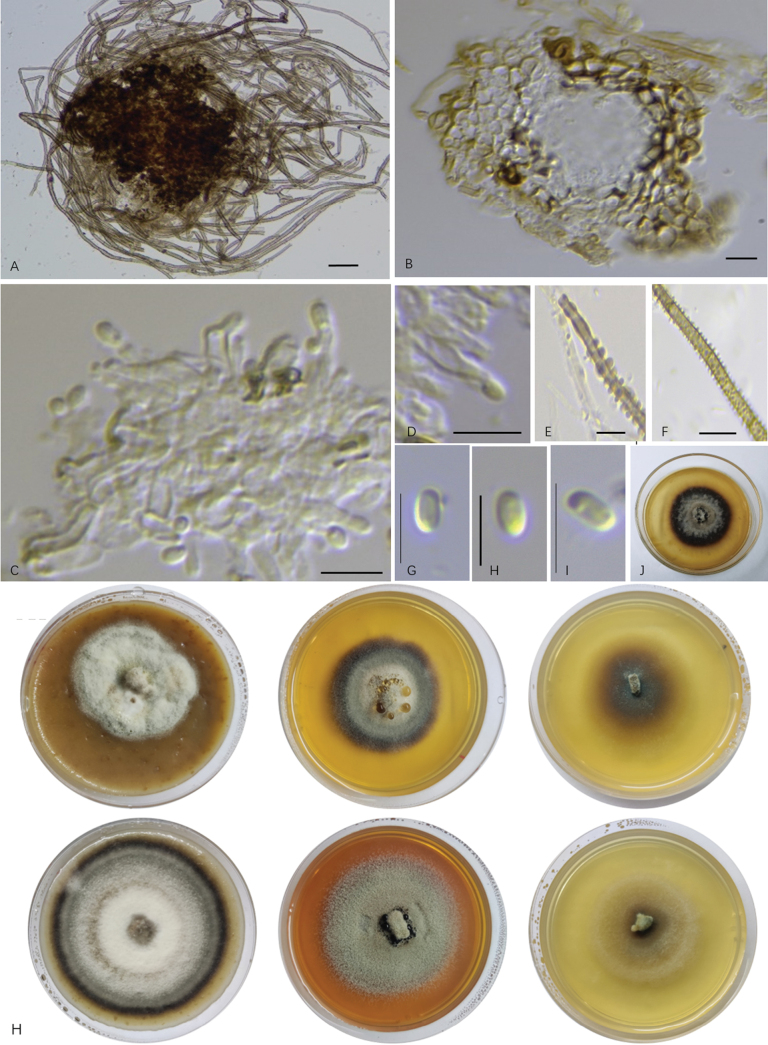
Morphological characteristics of *Nigromargaritatarda* in culures. **A** Conidiomata; **B** transect of conidiomata **C**; **D** conidiogenous cells; **E, F** verrucose hyphae; **G–I** conidia; **H** colony characteristics of *Nigromargaritatarda* in culture (upper: H331 at 73 days; bottom: H263 at 80 days; left: OA; middle: PDA; right: MEA); **J** oven-dried specimens of colony on PDA. Scale bars: 40 μm (**A**); 20 μm (**B, E, F**); 7 μm (**C, D, G–I**).

The colony diameters of H331 colonies were 1.5 cm, 1.1 cm and 1.5 cm for OA, PDA and MEA, respectively, after 7 days of culture at 27 °C in the dark. After 28 days, the colony diameters increased to 3.5 cm on OA, 2.6 cm on PDA and 3.4 cm on MEA, respectively. The colony on OA was superficial, circular with irregular margins, felt-like, white and brown in reverse. The colony on PDA was superficial, circular with regular margin, floccose to felt-like, greyish at the centre, brown at the margins and dark brown in reverse. The colony on MEA was submerged, brown with scarce aerial hyphae, circular in margin and brown in reverse (Fig. 2H).

The colony diameters of H263 colonies were 1.2 cm, 0.8 cm and 1.1 cm for OA, PDA and MEA, respectively, after 7 days of culture at 27 °C in the dark. After 28 days, the colony diameters increased to 4.0 cm on OA, 2.7 cm on PDA and 3.8 cm on MEA. The colony on OA was superficial, round, floccose to felt-like, central white, with wide concentric circles, regular dark brown margins and dark brown in reverse. The colony on PDA was superficial, circular in margin, greyish-green, velvety and dark brown in reverse, with brown pigmentation in the medium. The colony on MEA was superficial, circular at the margin, floccose to hairy, white to pale grey and light coloured in reverse (Fig. 2H).

##### Holotypus.

China • Gansu Province, Wuwei City, Minqin County; on healthy roots of *Gymnocarposprzewalskii*; September 2019; M. Li (Holotype, HMAS 352979; ex-type CGMCC3.27545).

##### Paratypus.

China • Gansu Province, Wuwei City, Minqin County; on healthy roots of *Gymnocarposprzewalskii*; July 2020; M. Li (Paratype, HMAS 352980; ex-paratype CGMCC3.27544).

### ﻿The introns Nta.18SS453 and Pcl Ple.18S1

**Figure 3.**
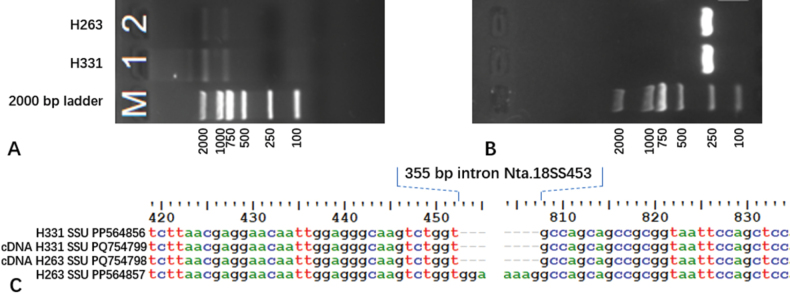
The amplification of intron Nta.18SS453 with rtPCR and its location on 18S rRNA gene. **A** The RNA of 28S (smaller than 2000 bp) and 18S (larger than 750 bp) rRNA were detected with gel electrophoresis (1.2% agarose, 120 V, 23 min); **B** the PCR product of 18S rRNA cDNA in H263 and H331; **C** cDNA alignment indicated the intron started following a “GT” and ended with an “AG” and was split off during transcription.

In the present study, an inserted DNA fragment of 355 bp in length was detected in the 18S region of H263. In order to validate whether the fragment is an intron of 18S RNA gene, the rtPCR was conducted to determine whether the fragment is cut out during 18S RNA gene transcription. With total RNA extracted from fresh mycelia of H331and H263, the 18S RNA and 28S RNA were visualised with gel electrophoresis (Fig. 3A) and the cDNA of 18S RNA were successfully amplified (Fig. 3B). Sequencing indicated the 355 bp DNA insertion in the 18S region of H263 was cut off in matured rRNA and, thus, was validated as an intron (Fig. 3C).

**Figure 4.**
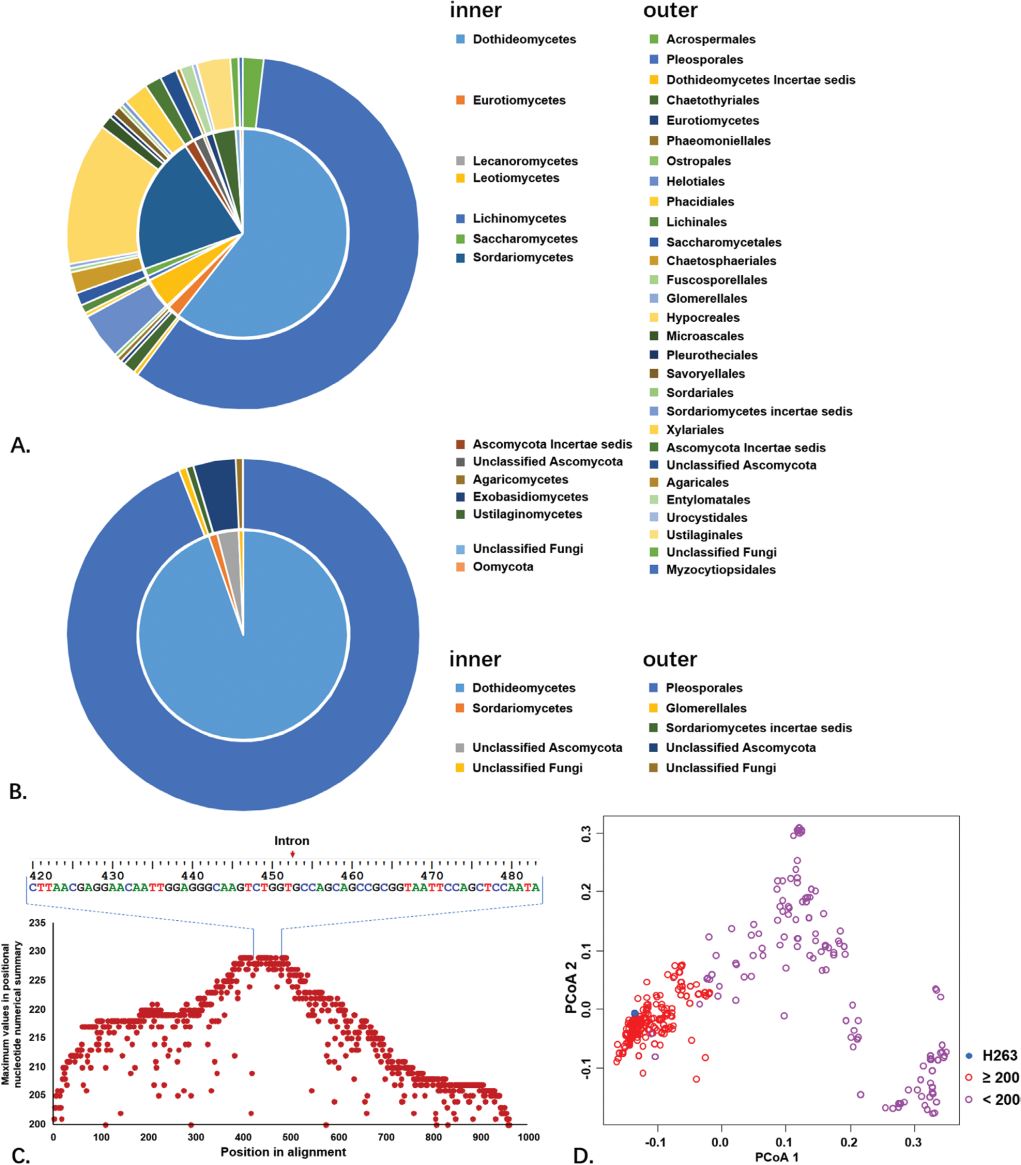
Brief information on intron Pcl Ple.18S1. **A** Taxonomic distribution of Nta.18SS453 and similar introns (dataset onlyintron_465); **B** taxonomic distribution of Nta.18SS453 and 287 references with alignment scores higher than 200; **C** the maximum number of positional nucleotides in the alignment of 229 sequences (dataset nointron_229), indicating conservation at each nucleotide. The consensus sequence at both flanks of the insertion is shown at the top; **D** PCoA plot showing dissimilarities in the alignment of 465 intron sequences (dataset onlyintron_465).

BLAST was employed to query 464 references with intron hits, using a maximum of 5,000 target sequences (Suppl. material 1). Derived from 259 taxa, these reference sequences were exclusively 18S genes, the majority of which belong to the Kingdom of Fungi, with the exception of *Oomycota* (Fig. 4A). Interestingly, when the BLAST hits were filtered to include only those with alignment scores of at least 200, 287 references remained, representing 152 taxa, 143 of which were *Pleosporales* (Fig. 4B, Suppl. material 1). One exception was a reference identified as *Colletotrichumgloeosporioides* (MH051114, *Glomerellales*). However, BLAST results suggested the MH051114 was misidentified (data not shown) and we excluded it from our analysis. The alignment of the nointron_229 dataset indicated that intron insertion occurred at highly-conserved sites in the 18S gene of all 229 references (Fig. 4C). Furthermore, the alignments of the 465 intron sequences suggested that the 287 high-scoring introns were more aggregated in PCoA plots than the low-scoring introns (Fig. 4D). This suggests that these introns are phylogenetically affiliated descendants from common ancestors and are specific to the 18S gene of *Pleosporales* species. Based on the principles proposed by Zhang and Zhang (2019), we propose the name Nta.18SS453 for the intron found in H263, referring to its presence at site 453 in the 18S gene of *N.tarda* for convenience of reference in future research. Moreover, we define the Pcl as Ple.18S1, which includes Nta.18SS453 and similar introns on the 18S gene of other *Pleosporales* members.

### ﻿The spread of Pcl Ple.18S1 within Pleosporales

**Figure 5.**
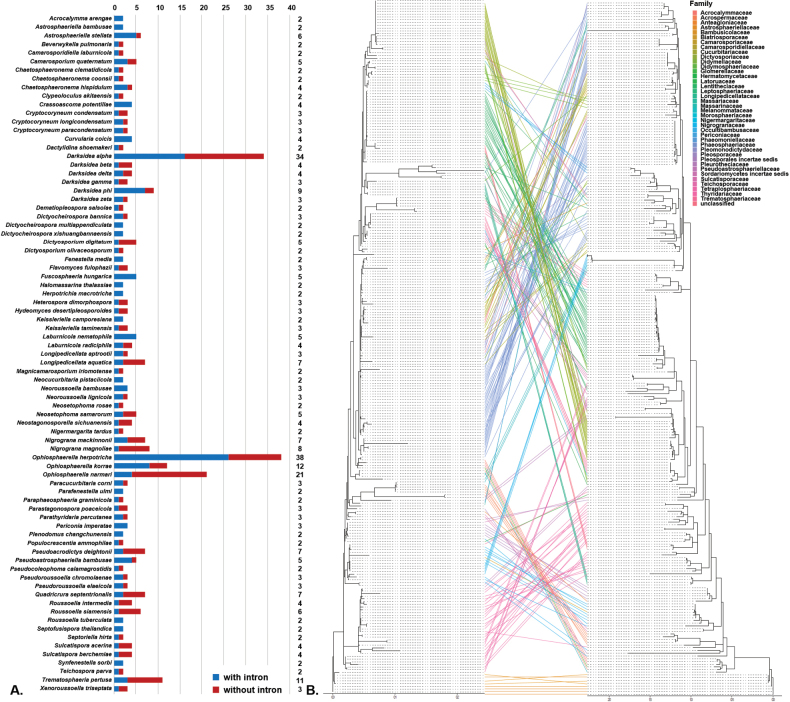
The Ple.18S1 intron distributions amongst fungal species. **A** Complete list based on all public sequences showing ratios between Ple.18S1 intron carriers and non-carriers within a certain species. The numbers in the right column indicate the numbers of 18S references deposited in the GenBank database; **B** co-phylogenetic comparison between ML trees in which 254 intron sequences and 254 18S sequences carried introns (left, dataset onlyintron_254; right, dataset nointron_254). Lines of different colours connect an intron and an 18S exon from the same reference in the GenBank database.

To investigate whether Ple.18S1 spreads amongst *Pleosporales* via horizontal (random jumping) or vertical (inheritance) transmission, we examined the distribution of Ple.18S1 amongst various species within the order. Our results indicated that Ple.18S1 introns frequently increased and, subsequently, decreased at the species level. Amongst the 126 introns detected, 80 species were found to have more than one 18S sequence deposited in GenBank, of which 60 species simultaneously possessed isolates with and without the intron (Fig. 5A). Despite the observed intraspecies incongruous distribution, parafit and PACo analyses revealed that the phylogeny of introns was significantly correlated with the phylogeny of the 18S gene (ParaFitGlobal = 226.94, *p* = 0.001 in parafit and *p* = 0 in PACo). In the comparison between ML trees, no obvious co-phylogeny pattern was observed at the families’ level amongst the host species of the introns (Fig. 5B). Nevertheless, frequent co-phylogeny was noted at lower taxonomical hierarchies, indicated by parallel connections.

**Figure 6.**
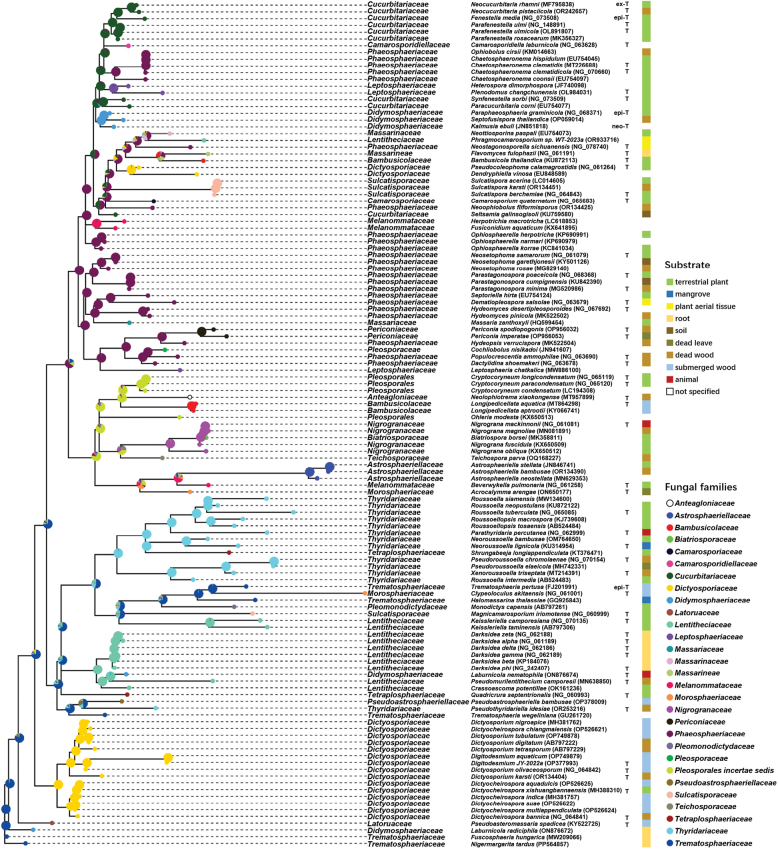
The ancestral state reconstruction of Ple.18S1 introns, showing their host alternation within *Pleosporales*, based on an unrooted ML tree (Suppl. material 2: RAxML_bipartitions.125_onlyintron_ML). The ancestral states are categorised into different families of intron carriers. Colours at each terminal node indicate the family of the intron host. Pie charts at each node indicate the relative likelihoods of ancestral states. Stacked colour chart approximately demonstrate the ecological traits of the habitats from which the fungi were recovered, according to the sequence information deposited in GenBank.

ASR provided further insight into the transmission dynamics of Ple.18S1 amongst host 18S genes. Fig. 6 illustrates the reconstruction of the host families of Ple.18S1 introns on an unrooted phylogenetic tree. The results revealed that the number of introns increased across most *Pleosporales* families. Introns hosted by *Phaeosphaeriaceae* formed polyphyletic clades, while, in families such as *Cucurbitariaceae*, *Dictyosporiaceae*, *Lentitheciaceae* and *Thyridariaceae*, most introns formed monophyletic clades, with a few exceptions, suggesting occasional interfamily host transitions. Some monophyletic clades were observed within genera, including Astrosphaeriella (Astrosphaeriellaceae), Longipedicellata (Bambusicolaceae), Periconia (Periconiaceae), Sulcatispora (Sulcatisporaceae), *Cryptocoryneum* and *Digitodesmium* (*Pleosporalesincertaesedis*). These findings suggest that Ple.18S1 introns have undergone vertical transmission along the host phylogeny to a certain degree, remaining conserved at the genus level.

## ﻿Discussion

### ﻿Morphological uniqueness of *Nigromargaritatarda*

*Nigromargaritatarda* were determined to *Pleosporales* in a preliminary sequence search. Although this taxon lacks teleomorphs, its pycnidia and aseptate, hyaline, simple conidia resemble the Phoma-like taxa in *Pleosporales* (Gruyter et al. 2013). Nevertheless, the most distinguished morphological feature of *N.tarda* is its ﻿exceptionally small pycnidia, typically ranging from 31 to 39 μm in diameter and never exceeding 50 μm in our study. This is in stark contrast to most other species within *Pleosporales*, which possess pycnidia or other types of conidiomata with diameters exceeding 100 μm (Zhang et al. 2012; Li et al. 2020). Even amongst Phoma-like genera, known for their pycnidia of varying sizes and hyaline, aseptate conidia, it is unusual to find pycnidia as small as those observed in *N.tarda* (Aveskamp et al. 2010; Chen et al. 2017). Several Phoma-like species with relatively small pycnidia include *Aposphaeriapulviscula* ((30–)54–100 μm diam., (56–)84–133 μm high), *Ascochytaneopisi* (80–120 µm diam., 80–100 µm high) etc. (Li et al. 2020). However, their pycnidia are still considerably larger than those of *N.tarda*. *Chaetophomaquercifolia* which possesses pycnidial conidiomata up to 30 µm in diameter according to Sutton (1980), is somewhat comparable to *N.tarda* in pycnidial size. However, the phialidic conidiogenous cells of *C.quercifolia* are ampulliform, whereas those of *N.tarda* are straight or curved cylindrical, presenting a morphological distinction. *C.quercifolia* is classified as *Pleosporalesincertaesedis* according to Li et al. (2020). Unfortunately, no sequence data for *C.quercifolia* have been published, precluding its inclusion in the phylogenetic analysis of *N.tarda*.

Members in *Trematosphaeriaceae* family can be divided to two ecological groups, viz. human dermatophytes, plant-associated fungi in marine habitats and root- associated fungi in terrestrial habitats. Nevertheless, this ecological divergence does not attribute to their phylogeny, as revealed in our analysis. In this study, multiple-locus phylogenetic analysis revealed that *N.tarda* represented an independent clade within the *Trematosphaeriaceae* under *Pleosporales*. This result also refuted *N.tarda* as any known Phoma-like taxa. Three genera in *Trematosphaeriaceae* have been established, based on sterile specimens, namely *Emarellia*, *Fuscosphaeria* and *Meanderella* (Borman et al. 2016; Pintye and Knapp 2021; Ahmed et al. 2022), while other members of the family produce septate ascospores. Unfortunately, *N.tarda* did not produce teleomorphic features in our study, preventing a direct comparison with other *Trematosphaeriaceae* members. Despite this, we still observed both similarities and distinct differences between *N.tarda* and other members of *Trematosphaeriaceae*. Several *Trematosphaeriaceae* members produced specialised hyphae structures, such as hyaline terminal chlamydospores in *Fuscosphaeriahungarica*, tuberculate in *Meanderellarijsii* and hyphopodia-like structures in *Trematosphaeriapertusa* (Suetrong et al. 2011; Pintye and Knapp 2021; Ahmed et al. 2022). *N.tarda* producespigmented, verrucous, thick-walled hyphae, a feature similar to *M.rijsii*. However, despite both being recovered from plant roots in arid ecosystems and sharing phylogenetic affinity, *N.tarda* is distinguished from *F.hungarica* with its pigmentation and unique morphological features of hypha, as well as its well-developed conidiogenous structures. Therefore, based on both morphological and molecular phylogenetic evidence, *N.tarda* forms an independent clade within the family *Trematosphaeriaceae* and represents a new fungal genus.

### ﻿The horizontal transmission of Ple.18S1 introns

Introns are prevalent in both fungal nuclear and mitochondrial genomes and have become important markers in phylogenetic and genome evolution studies of fungi (Matheny et al. 2006; Wang et al. 2020; Lücking et al. 2021). They are widely, though sporadically, distributed across fungal nuclear genomes and amongst different species, indicating frequent horizontal transfers and recurrent cycles of invasion, degeneration, loss and re-invasion, which highlight the dynamic nature of intron transmission (Reeb et al. 2007). For example, group I introns in the nuclear small-subunit ribosomal DNA (nuc-ssu-rDNA) have been gained independently on two separate occasions via horizontal transmission in mushroom-forming genera: once in the lineage leading to *Panellus* and once in the lineage leading to *Lentinellus* and *Clavicorona* (Hibbett 1996). Similarly, incongruent branching patterns between intron-based and rDNA-based phylogenies in *Sclerotiniaceae* suggested independent horizontal intron transfers (Holst-Jensen et al. 1999). The mechanisms for intra- and interspecies intron transfer include intron homing and reverse splicing. Intron homing occurs when introns containing homing endonuclease genes (HEGs) spread by recognising specific DNA sequences, thereby facilitating their insertion into intron-less alleles (Haugen et al. 2004). In addition, Bhattacharya et al. (2005) demonstrated that group I introns spread by reverse splicing in *Symbiotaphrinabuchneri*. In the present study, we identified a 355-bp intron, Nta.18SS453, in the 18S gene of *N.tarda*. Further analysis revealed that this intron belongs to the Pcl Ple.18S1 intron family, which is restricted to conserved insertion site within the 18S gene of *Pleosporales* species. It is, therefore, intriguing to explore the spread patterns and underlying mechanisms of this intron family within this fungal order.

The present study supports horizontal transmission as the primary mechanism for Ple.18S1 introns. The patterns in Fig. 5B and Fig. 6 indicated that the distribution of Ple.18S1 introns across *Pleosporales* families is phylogenetically uncorrelated, suggesting sporadic insertions in ancestral taxa of intron-rich families, such as *Cucurbitariaceae*, *Dictyosporiaceae*, *Lentitheciaceae*, *Phaeosphaeriaceae* and *Thyridariaceae*. In the ASR analysis, the ancestral nodes showing multiple potential states suggested that introns shifted hosts at the family level through horizontal transmission. Despite ecological data in GenBank references often being incongruous and incomplete, our analysis infers that this horizontal transmission is potentially related to specific habitats. For example, Ple.18S1 introns in *Trematosphaeriaceae* originated from two distinct sources: one transferred amongst fungi associated with healthy root (e.g. *Fuscosphaeriahungarica* from *Festucavaginata*, *Laburnicolaradiciphila* from *Triticumaestivum* according to GenBank and *Nigermargaritatardus* from *Gymnocarposprzewalskii*) and the other amongst fungi related to aquatic habitats (e.g. *Clypeoloculusakitaensis* from submerged woody plant, *Trematosphaeriapertusa* from a stump of *Fraxinusexcelsior* in a swamp and *Halomassarinathalassiae* from mangrove (Zhang et al. 2008; Suetrong et al. 2009). Accordingly, our analysis reveals that Ple.18S1 introns exhibit phylogeny affinity within similar habitats rather than within same family (*Trematosphaeriaceae*). In addition, inter-family intron horizontal transmission is demonstrated by multiple examples in Fig. 6, such as from *Cucurbitariaceae* to *Phaeosphaeriaceae* and *Leptosphaeriaceae* and from *Phaeosphaeriaceae* to *Massariaceae*, *Periconiaceae* and *Pleosporaceae*. Notably, Nta.18SS453 was detected in N.tarda H263, but absent in H331, which was collected from the same location and substrate. This observation suggests that H263 acquired the intron from an undetermined source in the root habitat, whereas H331 did not.

Considering BLAST screening failed to identify Ple.18S1 introns in organisms other than fungi and that the habitats where horizontal transmission occurred are ecologically distinct, non-fungal organisms, such as bacteria, may not mediate this horizontal transmission. Ple.18S1 introns lack endonuclease and occur exclusively at conserved loci within the 18S RNA gene rather than spreading across the genome. Therefore, they are unlikely to follow spreading mechanisms such as intron homing. One possible spread mechanism of Ple.18S1 introns is reverse splicing, as proposed by Bhattacharya et al. (2005), which plays a significant role in the intron spread in *Pezizomycotina* nuclear rDNA. Another minor possibility is RNA mediated transmission, similar to that observed in plants (Adams et al. 2000; Adams and Palmer 2003; Keeling and Palmer 2008).

### ﻿The vertical transmission and loss of Ple.18S1 introns

Beyond reverse splicing as a possible transmission mechanism, the interspecies transmission of Ple.18S1 introns within *Pleosporales* exhibited a phylogenetic pattern at low taxonomic hierarchies, such as the genus level. However, vertical transmission of Ple.18S1 introns from ancestral to descendant taxa during speciation is accompanied by frequent intron loss in certain species or strains (Fig. 5A). It has been suggested that fungi share a common ancestor with intron-rich genomes, yet massive intron reduction through intron loss has occurred in multiple fungal clades throughout their evolutionary history (Stajich et al. 2007; Lim et al. 2021). In the case of Ple.18S1 introns, a similar scenario is observed in *Darksidea*, a genus established in 2015, based on DSEs from semi-arid areas, which currently comprises nine species (Knapp et al. 2015; Romero-Jiménez et al. 2022; Tan and Steinrucken 2024a). Our results detected Ple.18S1 introns in six species of *Darksidea* with close phylogeny affinity, which suggested a vertical transmission from a common ancestor possessing the intron. However, while this observation may shift as more data accumulate, currently, no intron was detected in *D.epsilon*, *D.eta* and *D.theta*, which inhabit the same root habitat as other intron-carrying *Darksidea* species (Knapp et al. 2015; Tan and Steinrucken 2024a, 2024b). In addition, all six *Darksidea* species simultaneously possess strains that either carry or lack introns (Fig. 5A). This inter- and intra-species inconsistency of intron presence is likely attributed to intron loss. Therefore, frequent intron loss and gain events have been observed in our research, as in various fungal clades (Bolotin-Fukuhara and Fairhead 2014; Wu et al. 2021). The complex history of intron inheritance and lateral transfer within fungal rDNA implies that introns spread amongst genomes through various mechanisms (Bhattacharya et al. 2005).

### ﻿Host range restriction of intron Pcl Ple.18S1

It is well established that certain fungal introns are restricted to specific taxonomic or phylogenetic groups. For example, Holst-Jensen et al. (1999) reported that introns 788SSU and 798LSU were found exclusively within members of *Helotiales*. In a survey of introns in 39 mushroom-forming species, introns were confined to the genera *Punellus*, *Cluvicoronn* and *Lentinellus*, suggesting a pattern of horizontal transmission followed by vertical inheritance within these lineages (Hibbett 1996). One compelling question that arises is why *Pleosporales* specially accommodates Ple.18S1 members.

Beyond the genetic and biochemical mechanisms underlying horizontal transmission, the biological and ecological properties responsible for bringing the two taxa close enough are also essential (Holst-Jensen et al. 1999). *Pleosporales* is a fungal order closely associated with plant materials. Most of the *Pleosporales* reference sequences in our analysis were sourced from plant matter, including living or dead leaves, twigs, stems and roots; decaying litter; submerged wood; and soils under vegetation, many of which belong to DSEs. These habitats, characterised by temporal or spatial proximity, may provide ample opportunities for horizontal intron transfer amongst *Pleosporales* members.

## ﻿Conclusion

In the present study, we described a new fungal taxon within *Trematosphaeriaceae*, *Nigromargaritatarda* gen. et sp. nov., which inhabited the roots of desert plant *Gymnocarposprzewalskii* as a dark septate endophyte in China. Notably, one strain of *tarda* carries a 355 bp intron in the 18S gene, which belongs to an intron position class (Pcl) restricted to species within the order *Pleosporales*. The intron and Pcl were named as Nta.18SS453 and Ple.18S1, respectively. Our findings suggest that Ple.18S1 introns primarily spread amongst *Pleosporales* families through horizontal transmission, likely via reverse splicing. This horizontal transmission appeared to occur in specific habitats, particularly root and aquatic environments. Additionally, Ple.18S1 introns underwent loss and reduction during vertical inheritance from ancestral to descendant species. This study established a new genus in *Trematosphaeriaceae* and depicted the spread features of a less-documented Pcl amongst *Pleosporales* families with high resolution. By analysing the distribution and phylogenetic relationships of Ple.18S1 introns, we provide valuable insights into the evolutionary processes in *Pleosporales* and the mechanisms of intron transmission contribute to the intricate phylogeny of these fungi. Future studies focusing on the functional roles of these introns and their potential ecological implications will be essential to further elucidate their evolutionary significance in fungal biology.

## Supplementary Material

XML Treatment for Nigromargarita

XML Treatment for Nigromargarita tarda

## ﻿Additional information

### Adherence to national and international regulations

All the fungal strains used in this study have been legally obtained, respecting the Convention on Biological Diversity (Rio Convention).

### Author ORCIDs

Meng-Yuan Li https://orcid.org/0009-0005-7352-7991

Xiang Sun https://orcid.org/0000-0002-6875-4971

Yu-Qing Liu https://orcid.org/0009-0005-2544-9394

Sheng-Hui Qin https://orcid.org/0009-0005-3095-8046

Min Li https://orcid.org/0009-0001-8215-8906

Xue-Li He https://orcid.org/0000-0002-2783-3390

### Data availability

The sequence data generated in current research have been deposited in GenBank and all datasets are provided in supplementary files.

## Supplementary materials

Supplementary material 1 Detailed information and alignment matrices for all datasets Data type docx https://binary.pensoft.net/file/1265942This dataset is made available under the Open Database License (http://opendatacommons.org/licenses/odbl/1.0/). The Open Database License (ODbL) is a license agreement intended to allow users to freely share, modify, and use this Dataset while maintaining this same freedom for others, provided that the original source and author(s) are credited.Meng-Yuan Li, Xiang Sun, Yu-Qing Liu, Sheng-Hui Qin, Min Li, Xue-Li He

Supplementary material 2 ZIP-archive Data type zip Explanation note The detailed information and alignment matrices for datasets listed in Suppl. material 1, as well as tree files generated and R codes used through the paper. https://binary.pensoft.net/file/1265943This dataset is made available under the Open Database License (http://opendatacommons.org/licenses/odbl/1.0/). The Open Database License (ODbL) is a license agreement intended to allow users to freely share, modify, and use this Dataset while maintaining this same freedom for others, provided that the original source and author(s) are credited.Meng-Yuan Li, Xiang Sun, Yu-Qing Liu, Sheng-Hui Qin, Min Li, Xue-Li He
