# Supplementary material for: ﻿Nigromargaritatarda gen. et sp. nov. and distribution of an intron position class within Pleosporales
Source: IMA Fungus. 2025 Feb 28;16:e145425. doi: 10.3897/imafungus.16.145425 (PMC11889510; doi:10.3897/imafungus.16.145425)
Supplement: Supplementary material 1 — Detailed information and alignment matrices for all datasets [file imafungus-16-e145425-s001.docx]

| **Table S1** Detailed information and alignment matrices for all datasets | | |
| --- | --- | --- |
| Dataset | Sequence information | Alignment matrix |
| combine_60 | See Table 2 | combine_60.fasta |
| onlyintron_465 | onlyintron_465.xlsx | onlyintron_465.fasta |
| all_254 | Co-phylogeny-254.xlsx | all_254.fasta |
| onlyintron_254 | Co-phylogeny-254.xlsx | onlyintron_254.fasta |
| nointron_254 | Co-phylogeny-254.xlsx | nointron_254.fasta |
| nointron_229 | nointron_229.xlsx | nointron_229.fasta |
| species_ratio | ratio.xlsx | - |
| ASR_ onlyintron_125 | ASR_ onlyintron_125.xlsx | ASR_onlyintron_125 |
